# C-755-01. Non-tobacco Nicotine Dependence Increases Risk of Wound Complications, Mortality, and Opioid Use Disorder Following Burns

**DOI:** 10.1093/jbcr/irag033.037

**Published:** 2026-04-06

**Authors:** Matthew Q Dao, Anika Y Kim, Sarah Wang, Paul Won, Eloise W Stanton, Haig A Yenikomshian, Maxwell B Johnson

**Affiliations:** University of Texas Medical Branch - John Sealy School of Medicine, Galveston, TX; Division of Plastic and Reconstructive Surgery, Keck School of Medicine, University of Southern California, Los Angeles, CA; Keck School of Medicine, University of Southern California, Los Angeles, CA; Division of Plastic Surgery, Department of Surgery, McGovern Medical School, University of Texas Health Science Center at Houston, Houston, TX; Division of Plastic and Reconstructive Surgery, Keck School of Medicine, University of Southern California, Los Angeles, CA; Division of Plastic and Reconstructive Surgery, Keck School of Medicine, University of Southern California, Los Angeles, CA; Division of Plastic and Reconstructive Surgery, Keck School of Medicine, University of Southern California, Los Angeles, CA

## Abstract

**Introduction:**

Nicotine delivery from non-tobacco products (e.g., electronic cigarettes, patches, pouches, gums, lozenges, and oral sprays) is becoming increasingly prevalent. While cigarettes and tobacco products are known risk factors for impaired wound healing, the isolated effects of non-tobacco nicotine dependence (NTND) on post-burn recovery are unclear. The present study sought to assess the impact of NTND on short and long term complications following burn injury.

**Methods:**

We conducted a retrospective cohort analysis through the United States Collaborative Network on TriNetX, a federated database. Adult burn patients (18+ years) with NTND were compared to burn patients without any nicotine dependence. Patients with a documented history of cigarette smoking or tobacco product usage were excluded. Propensity score matching (1:1) was performed for demographics, body mass index, comorbidities, concurrent substance use disorders, burn characteristics (region of bodily injury, total body surface area affected, and degree of burn), and treatment modalities (non-steroidal anti-inflammatory medications, autografts, and debridements). Primary outcomes at 90 days were wound complications (infection, disruption, and hematoma formation), sepsis, and healthcare utilization (antimicrobial prescriptions and emergency department [ED] visits). Secondary outcomes were mortality and new diagnosis of opioid use disorder within 1 year. Associations were quantified using risk ratios (RRs), with statistical significance set at *p*<.05.

**Results:**

An unmatched total of 594 308 burn patients were identified (21 050 with NTND; 573 258 controls). After 1:1 matching, 20 906 patients remained in each cohort. Within 90 days post-burn, the matched NTND patients had significantly higher risk of wound disruption (RR 1.81, *p*=.004), wound infection (RR 2.65, *p*<.001), sepsis (RR 1.50, *p*=.003), antimicrobial prescriptions (RR 1.35, *p*<.001), ED visits (RR 1.51, *p*<.001), and mortality (RR 1.20, *p*=.033) when compared to matched controls. Risk of hematoma formation was higher in the NTND cohort, though not statistically significant (RR 1.54, *p*=.193). Within 1 year after burn injury, patients with NTND had increased risk of mortality (RR 1.20, *p*=.002) and opioid use disorder (RR 4.24, *p*<.001) compared to those without nicotine dependence.

**Conclusions:**

Non-tobacco nicotine dependence was significantly associated with elevated risk of complications, healthcare utilization, and long-term mortality as well as onset of opioid use disorder after burn injury. Given these findings, nicotine, independent from tobacco, may impair immune function and tissue healing, which could exacerbate burn prognosis.

**Applicability of Research to Practice:**

Burn care providers are encouraged to screen patients for regular use of non-tobacco nicotine products upon admission and offer cessation counseling, as addressing this risk factor may reduce complications and improve recovery.

**Funding for the Study:**

N/A.

